# Proof-of-concept validation and characterisation of a DLP 3D bioprinted vascularised colorectal cancer model

**DOI:** 10.3389/fbioe.2026.1944341

**Published:** 2026-10-01

**Authors:** Atanas Valev, Yordan Sbirkov, David Liebl, Eva Ďurinová, Daniela Popelková, Aleš Benda, Michael Ausserlechner, Murad Redzheb, Victoria Sarafian

**Affiliations:** 1 Department of Medical Biology, Medical University of Plovdiv, Plovdiv, Bulgaria; 2 MatriChem, Sofia, Bulgaria; 3 Research Institute, Medical University of Plovdiv, Plovdiv, Bulgaria; 4 Imaging Methods Core Facility at BIOCEV, Faculty of Science, Charles University, Prague-West, Czechia; 5 Department of Pediatrics I and 3D Bioprinting Lab, Medical University of Innsbruck, Innsbruck, Austria

**Keywords:** channels, colorectal cancer, DLP bioprinting, GelMA, HCT-116, HUVEC, vascularisation

## Abstract

Tumor vascularisation is essential in promoting cancer growth and metastasis and represents a key target for efficient drug delivery. Nevertheless, *in vitro* experimental models are limited to monolayer (co)cultures of endothelial and malignant cells, scratch and transwell migration assays where the vascularisation process is largely neglected. Direct contacts between different cell types and extracellular matrix (ECM) components, and processes like cell migration and invasion and ECM remodeling are also difficult to reproduce using conventional culturing methods. This study aims to address some of these limitations by developing digital light processing (DLP) 3D bioprinted model of colorectal cancer based on methacrylated gelatin (GelMA) and containing endothelialised channels. The materials and approaches proposed here were additionally validated with 3D biofabricated in-house made designs based on the (bio)casting of GelMA and collagen hydrogels, and with 3D printed transwell assays. We found that our DLP bioprinting and 3D biofabrication protocols support the growth of HCT-116 colorectal cancer cells (embedded in the gel) and the survival of HUVEC endothelial cells (seeded to the channels), that cells adhere well to the collagen and GelMA matrices, and that the cells can remodel the hydrogel as revealed by scanning electron microscopy (SEM). Importantly, we also found that HUVECs migrate through these substrates in both the channel and transwell constructs. Altogether, to our knowledge, this pilot study presents the first DLP bioprinted model of CRC using GelMA, and the only DLP bioprinted colorectal cancer model seeded with HUVECs suitable for *in vitro* study of tumor vascularisation and drug delivery.

## 1 Introduction

Metastases ultimately kill about 90% of cancer patients (Fares et al., 2020). For example, in metastatic colorectal cancer, which has a rising incidence in developing countries, the 5-year overall survival is merely 13%. The critical role of angiogenesis in tumor growth, invasion and cell spreading to distant parts of the body has been well established (Liu et al., 2023). However, our understanding of the interfaces between cancer and endothelial cells is largely limited due to the lack of physiologically relevant *in vitro* experimental systems.

New experimental strategies like organ-on-a-chip provide more intricate experimental systems with well-defined spatial organization, but their perfusion usually occurs through artificial (plastic) microchannels, rather than through engineered vascularised cell-permeable channels surrounded by a tumor mass. Current 3D bioprinting technologies and available biomaterials are still searching for optimal combinations that allow for high-resolution printability without compromising biocompatibility.

To date, the DLP bioprinting allows for the best potential resolution (10–50 µm in x-y-z axes) among the main types of 3D bioprinting (extrusion-based and inkjet being the other two widely used and affordable methods). Nevertheless, there are very few examples of its use for mimicking vascularisation (in contrast to DLP cell-free 3D printing). A recent study demonstrated that DLP bioprinting with gelatin-norbornene (GelNB) (2%–5%) and PEG4SH allows for the sprouting of endothelial cells from channels (300–1,200 µm in diameter) into the bulk of the gel (Duong and Lin, 2023). A similar approach with PEG/GelMA perfusable channels seeded with HUVECs and bulk containing both endothelial cells and MSCs was used in an *in vivo* neovascularisation mouse model where blood vessel formation within the channels could be observed as early as 2 weeks after the implantation demonstrating the physiological relevance of this type of models (Parkhideh et al., 2023). Paone et al. (2023) have developed a PEG norbornene-based blood brain barrier model where astrocytes are printed in a construct with channels that are seeded with endothelial cells demonstrating that DLP bioprinting is applicable for functional and perfusable vascularised *in vitro* models (Paone et al., 2023).

Based on our previous experience with 3D bioprinting and cancer modeling (Sbirkov et al., 2021; McGuckin et al., 2023) and with hydrogel development and implementation (Gospodinova et al., 2021), here we designed a novel model to mimic tumor vascularisation. The proposed experimental platform combines 3D digital light processing (DLP) bioprinting and two different cell types (colorectal and endothelial) spatially organised into a tumor bulk and endothelialised channels that span the “tumor mass”. The aim of this study was to develop and validate an accessible and reproducible experimental platform for endothelial cell-cancer cell interactions with different biomaterials that allow for representative *in vitro* models for basic research and for drug screening.

Therefore, to date and to the best of our knowledge, this is the only/first cancer model with channels seeded with HUVEC cells that is DLP bioprinted.

## 2 Materials and methods

### 2.1 Hydrogel and resin preparation

Printing and bioprinting resins were prepared via specifically designed protocols by MatriChem LTD. The main prepolymer used was methacrylated gelatin (GelMA) produced by MatriChem Ltd., with 94% degree of substitution. Lithium phenyl-2,4,6-trimethylbenzoylphosphinate (LAP, Biosynth) and Tartrazine were used as photoinitiator and photoabsorber, respectively. The final formulation used for bioprinting and other methods of biofabrication of cell-laden GelMA gels (see below) was selected as 10% GelMA with 5 mg/mL LAP and 0.5 mg/mL Tartrazine. Additionally, a 1% neutralized solution of Type I collagen (product of MatriChem Ltd.) was used to cast cell-laden hydrogels to validate the experimental strategy (see below) and for sealing of entry/exit ports of channels during seeding.

### 2.2 Cell culture and 3D bioprinting and casting

HCT-116 cells were cultured in standard conditions - DMEM with 10% FBS and 0.5% Pen/Strep (all purchased from PanBiotech, Germany). Cells were harvested and pelleted down, supernatant was aspirated and cells were resuspended to yield a cell-laden resin of 0.5 × 10^6^ cells/mL. This concentration of HCT-116 cells were used for the biocasting of channels as well. Several resin formulations were tested together with various extraction/synthesis/preparation methods (referred to as batch#1 and batch#2) and optimisation of different printing conditions was carried out (see Materials and methods). For this set of experiments, discs (5 mm in diameter and 300 µm in thickness) (Supplementary Figure S1A and See Section 3.1) were bioprinted using the GelMA-based resins with different concentrations of GelMA (5% and 10%) and a constant density of HCT-116-cells (5 × 10^5^ cells/mL). The discs were produced in triplicates for each of three cross-linking times that were dependent on the resin’s GelMA concentration –1X, 2X and 4X resin’s minimum cross-linking time as determined in dedicated validation procedures during manufacturing (corresponding to 20, 40 and 80 s for 5% GelMA and 10, 20 and 40 s for 10% GelMA). The bioprints were cultured for 12 days in DMEM +10% FBS. At each timepoint on day 1, 4, 6 and 12, 3D DLP bioprints were stained with calcein AM and propidium iodide to determine cell viability. Percentage live cells was calculated after manual counting of Calcein and PI positive cells from at least four fields of view of 0.5 × 0.5 mm from at least two separate bioprint images. Student’s t-test was carried out to compare between different crosslinking times in order to determine the optimal resins and conditions for sufficient cell survival. HUVEC/hTert-EYFP cells (referred to as just HUVECs here) were kindly provided by Prof. Michael Ausserlechner (Medical University of Innsbruck, Austria) and cultured as previously described [9].

### 2.3 3D bioprinting and biofabrication designs

Hydrogel printing and bioprinting was performed on a Elegoo Mars 4 DLP resin 3D printer modified with a source (AU-3D Printer, Auervo-UK) and a passive system for maintaining humidity. Samples were printed on silylated (3-(Trimethoxysilyl)Propyl methacrylate, 440159, Sigma) 22 × 22 mm glass coverslips (according to a previously published protocol (Underhill et al., 2007) affixed to the build plate. Cell-free prints of a serpentine channel (6 parallel sections with rounded bends in a 10 × 10 × 2 mm block, 1 mm in diameter, ∼50 mm total length) were produced from GelMA-PEGDA and used to validate cell adhesion. Those channels were then coated with Poly-L-Lysine, seeded with 20 × 10^3^ HUVEC-YFP cells (kindly provided by Prof. Michael Ausserlechner at the Medical University of Innsbruck). The design for the next set of experiments was simplified to a block (15 × 7.5 × 2 mm) containing two parallel channels of 1.2 mm diameter, spaced 1 mm from each other (Supplementary Figure S1B and See Section 3.2).

As an alternative method to validate the cell adhesion in channels independently of the 3D printing process and the material used, a protocol for casting of both cell-free and cell-laden hydrogels was used to generate analogous structures from the same GelMA-based resin used for bioprinting and from 1% collagen solution. The material was pipetted in a specially designed and 3D printed 10 × 25 × 2 mm cast/mold (See Section 3.4). The cast was sandwiched between two 22 × 22 mm coverslips and two rods were inserted in order to form two parallel channels (subsequently sealed with collagen).

In the case of the GelMA resin, the cast was illuminated by the printer for a length of time equal to the total amount of time for each layer of the equivalent print (300”), then flipped and illuminated for the same length of time from the other side. For collagen, the print was left for 1 h in an incubator at 37 °C to cross-link thermally. Identical casting methodology was implemented for creating disk-shaped bottoms of in-house designed and 3D printed transwell inserts (Supplementary Figure S1C).

### 2.4 Cell seeding

Coating of channels was done with 0.1 mg/mL Poly-L-lysine (P4707, Sigma-Aldrich, United States) for 30 min at 37 °C followed by a single wash with ∼2 mL of PBS. The Poly-L-lysine-coated (and for initial optimisations also uncoated channels to compare to) were seeded with ∼40 µL containing a total of ∼20 × 10^3^ HUVECs per channel which corresponds to a seeding density of 1,000 cells/mm2. To prevent leakage and evaporation during the subsequent incubation period, the channel entry/exit ports were sealed with 1% collagen that was thermally cross-linked for 15 min at 37° C. Both the cell-free prints and the 3D bioprints with channel were mounted into rectangular 3D printed casts, similar to the one used for casting of GelMA and collagen, in order to be fixed into position in 50 mL falcon tubes. For 3D printed channels, sterile tissue soaked with PBS was left at the bottom of the tube to maintain humidity, while for 3D bioprinted channels tubes were filled with DMEM. The falcons were then placed in a rotator (2 rpm) inside a cell culture incubator overnight. For transwell assays aiming to demonstrate the suitability of hydrogels and resins to support HUVEC adhesion and migration, ∼20 × 10^3^ HUVEC cells were seeded on top of each insert.

### 2.5 Rheology

Oscillatory shear rheology was used to characterise the viscoelastic properties of the 5% and 10% GelMA hydrogels (as the ones showing best biocompatibility for 3D bioprinting). A shear stress amplitude of 1 Pa was used to evaluate storage modulus (G’) values. To ensure that the reported moduli would reflect intrinsic network properties rather than nonlinear yielding effects (Ribeiro et al., 2017), this stress level was selected from the low-stress regime of a controlled oscillatory stress ramp up to 200 Pa.

## 3 Results

### 3.1 Optimisation of GelMA 3D bioprinting of CRC cells

The first objective was to establish a balance between biocompatibility and printability (mechanical properties that could support fine structures like channels) of GelMA. For this purpose, two resin formulations–batch#1 and batch#2, at two concentrations of GelMA (5% and 10%) and three curing times each were tested (see Materials and methods). Out of these 12 conditions, we found that3 conditions supported continuous proliferation of cancer cells (short and intermediate cross-linking time for batch#2 5% GelMA and intermediate cross-linking time for batch#2 10% GelMA - Figure 1). Nevertheless, the 10% GelMA was selected as a prime candidate for further bioprinting steps since the 5% resins, especially at short cross-linking times, produced gels with poor printability and mechanical properties.

**FIGURE 1:**
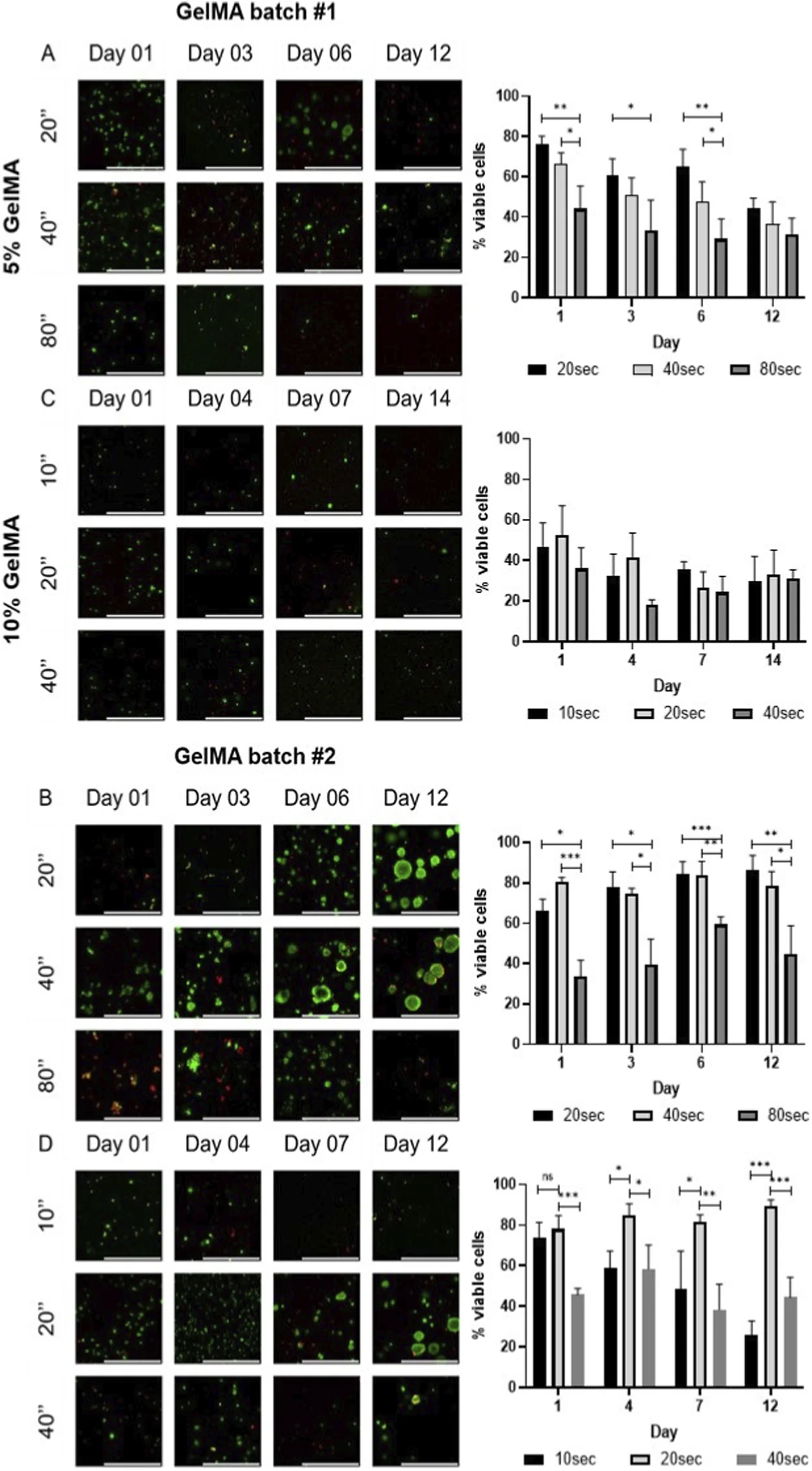
Assessment of GelMA biocompatibility with HCT-116 CRC cells. Staining with calcein AM/PI for live/dead cells of bioprinted HCT-116 cells in a 5% resin of GelMA, batch#1 **(A)** or batch#2 **(B)**; bioprinted HCT-116 cells in a 10% resin of GelMA, batch#1 **(C)** or batch#2 **(D)** at annotated timepoints. Scale bar: 500 µm. Percentage live cells depicted on the right for each of the four resins. Error bars represent mean with SD of at least four data points. Student’s t-test indicating significance between percentage viable cells in resins with different crosslinking time as annotated (*p ≤ 0.05, **p ≤ 0.005, ***p < 0.0005, ns: not significant).

Rheological studies also supported the biological findings that 10% GelMA would be more appropriate. An increase in stiffness from 5% GelMA (G’∼0,12 kPa) to 10% GelMA (G’∼1,8 kPa) was observed (Supplementary Figure S2). This is consistent with the high degree of methacrylation of the GelMA used here (94% relative to the phenylalanine peak; 0.479 mmol g^-1^ by TMSP-based NMR), which considerably increases effective crosslink density.

Subsequent cell-free pilot 3D printing experiments confirmed that sub-milimeter channels can be successfully printed from 10% GelMA (Figure 1).

### 3.2 Optimisation of 3D bioprinting of channels and endotheliasation (seeding)

After determining DLP bioprinting conditions supporting cell proliferation and providing sufficient mechanical properties of the product, we focused on optimisation of the printing process and cell-seeding of the channels (Figure 2). Two designs were used and tested–one with serpentine channel (Figure 2 – left) and one with two straight parallel channels (Figure 2 – right), both with a diameter of 1.2 mm. Application of static incubation of cell-seeded gels for a defined time (e.g. 30 min) followed by manual flipping of the gels for another round of seeding (method reported earlier by multiple groups (Hwang et al., 2024; Cai et al., 2025; Maggiotto et al., 2025) resulted in gels with clumps of unevenly distributed cells. We therefore adopted a seeding strategy relying on slow constant rotations (see Materials and methods) and tested several seeding times allowing for cells to adhere evenly on the surface of the channels. We also found that seeding cells overnight on a rotator placed within a standard cell culture incubator (Figure 2 bottom) was the most efficient.

**FIGURE 2:**
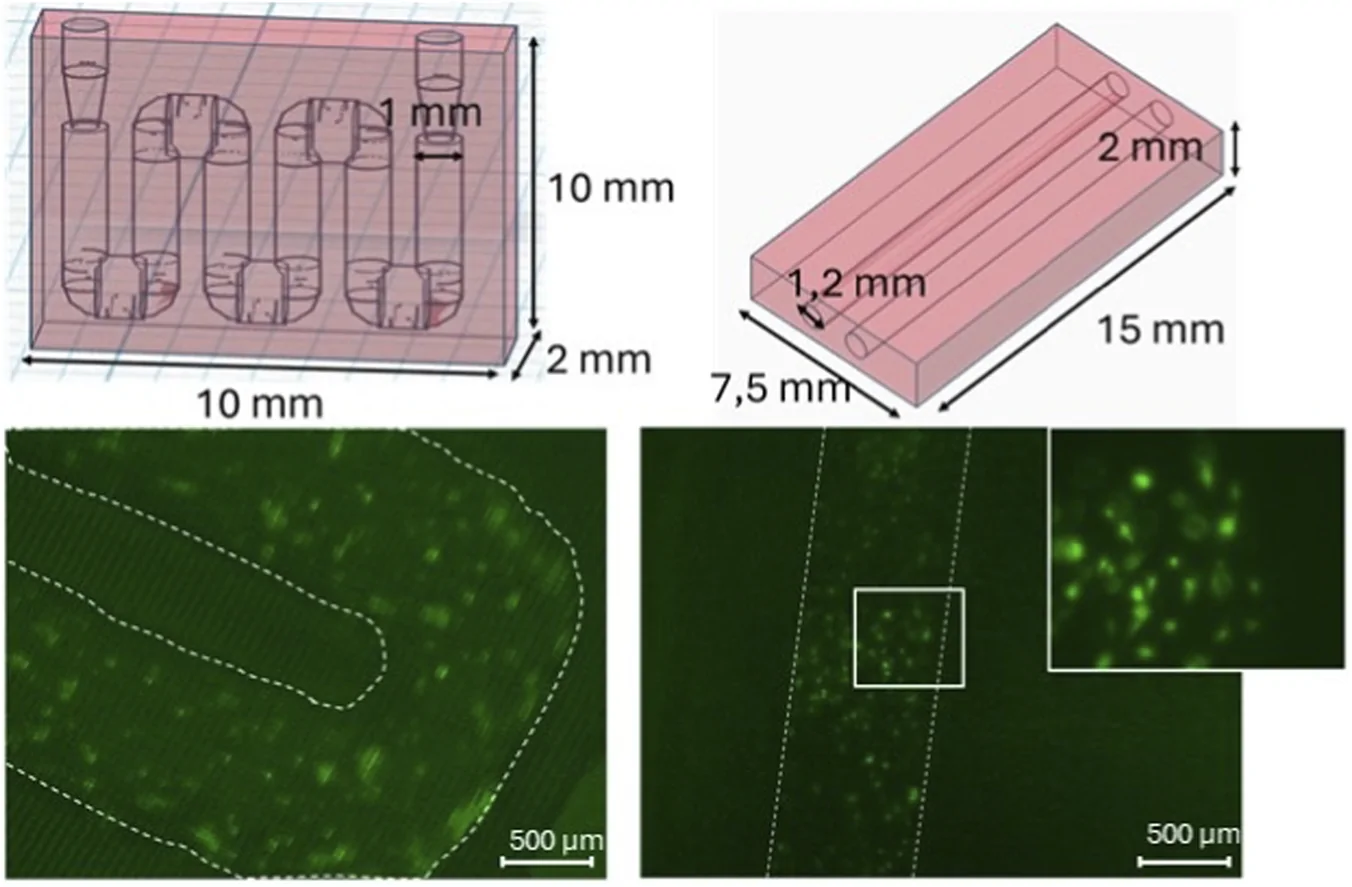
Design of DLP 3D printed channels and validation through seeding with HUVEC cells. Top: 3D design of serpentine channels (left) for seeding optimizations and parallel channels (for HUVEC migration experiments). Bottom: Fluorescent microscopy pictures of HUVECs distributions along the channels 24 h after seeding.

The homogenous distribution of cells throughout the channel was confirmed by confocal microscopy (Z-stack as Supplementary Methods and Supplementary Figure S1E). This seeding method was further validated and reproduced through two biocasted models with GelMA and collagen.

### 3.3 Characterisation of cell-matrix interactions, cell growth and migration through the gels

After optimising GelMA formulations and cell seeding of channels, we proceeded with the 3D bioprinting of HCT-116 cells in the similar format and design with channels to be subsequently seeded with HUVECs. Major technical challenge we encountered was that HUVECs proliferated in the channels only up to 4–5 days of cultivation before they detached and died, likely from hypoxia or local depletion of growth factors (cultivation without perfusion), which has been demonstrated by previous studies with and without perfusion of endothelialised channels (Osaki et al., 2015; Funamoto et al., 2017). In order to enhance cell migration within relatively limited time window of HUVEC cell viability inside the channels, we only used the gel design with two parallel channels whereby one was seeded with HUVEC cells and the other perfused with VEGF to stimulate HUVEC cell migration between the channels. Thus, after a 24 h stimulation with VEGF, the DLP bioprints were fixed and characterised via electron microscopy (SEM) and immunohistochemistry (IHC) (Figure 3 and Supplementary methods).

**FIGURE 3:**
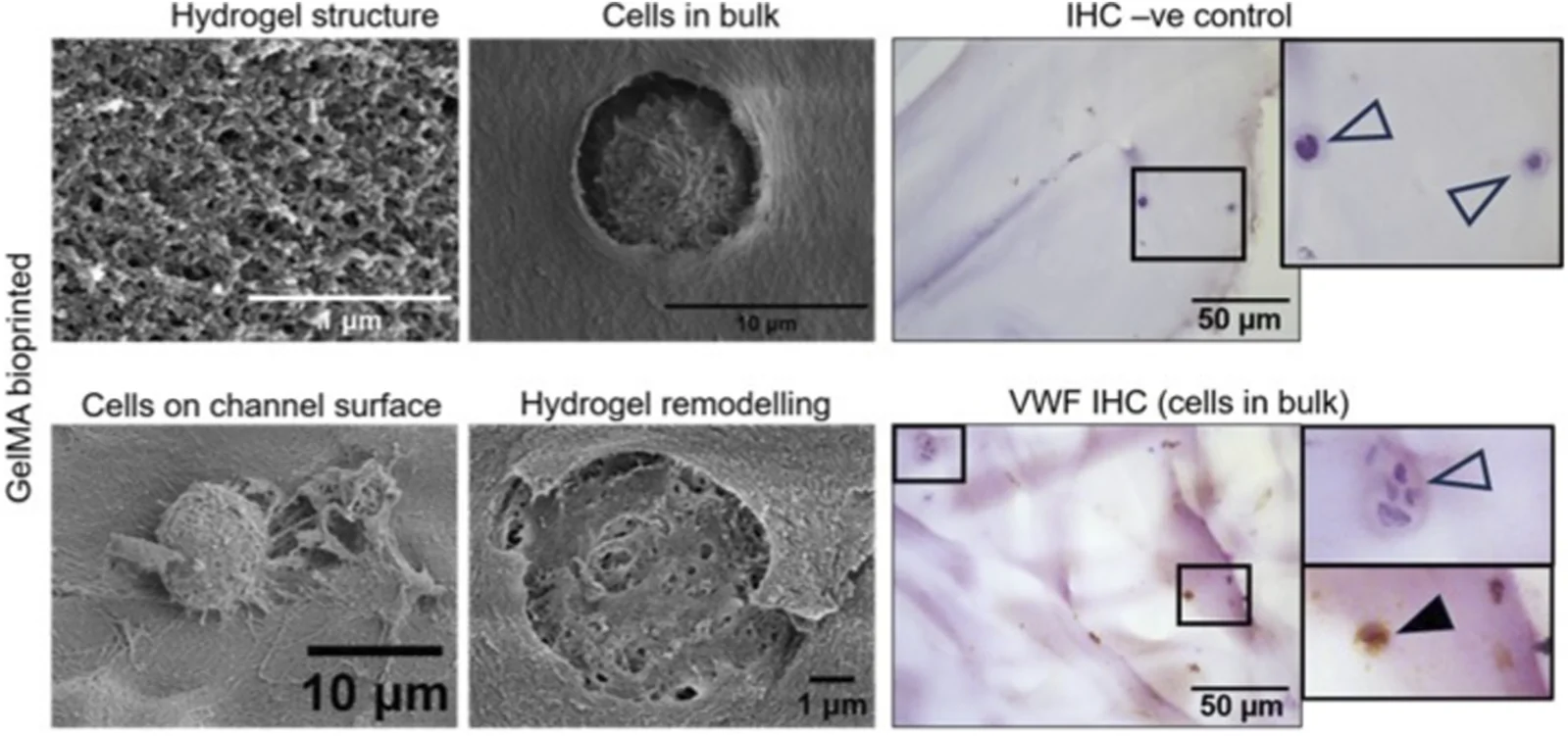
Characterisation of hydrogel-cell interactions. Left: SEM images showing hydrogel structure, cells embedded in the hydrogel or on the surface of the channel, and remodelling of the hydrogel by the cells. Right: IHC representative images at ×40 magnification for staining of endothelial cells with an anti-VWF antibody. Cells that are positive for VWF and can be identified as HUVECs are annotated by black arrowhead, while VWF-negative cells are stained only with hematoxylin and are annotated by open arrowheads (negative control top right, IHC staining bottom right).

Besides gaining a valuable insight into the structure and porosity of the 10% GelMA, we also observed several key cell characteristics. SEM images demonstrated that cells make direct contacts with the substrate and, importantly, can remodel the hydrogel, which is a critical property of the gel that would allow for the migration of cells (Figure 3 bottom left). Positive detection of the von Willebrand factor (an endothelial marker) in cells localised in the bulk of the bioprints, rather than in the lumen of the channel, demonstrated that at least some HUVECs migrated through the 10% GelMA (Figure 3 bottom right). These observations provided a proof-of-concept for the feasibility of this model to study angiogenesis or endothelial cell sprouting.

### 3.4 Validation of biocompatibility, seeding strategies, and transmigration of HUVECs through novel blueprints for tumor vascularisation modeling

Finally, we tested another set of 3D biofabrication strategies to extend the validation of 10% GelMA biocompatibility and the ability of HUVECs to migrate throughout the gel. For this we employed 3D printing of molds with subsequent (bio)casting of HCT-116-laden GelMA (using collagen as a parallel control) with formation of channels that can also be seeded with endothelial cells (Figure 4). The results (Supplementary Figure S3) were consistent with those for the DLP 3D bioprinted model, confirming biocompatibility of the gel, formation of cell-substrate contacts and HUVECs migration from the channel into the bulk of the gel within a couple of days upon seeding.

**FIGURE 4:**
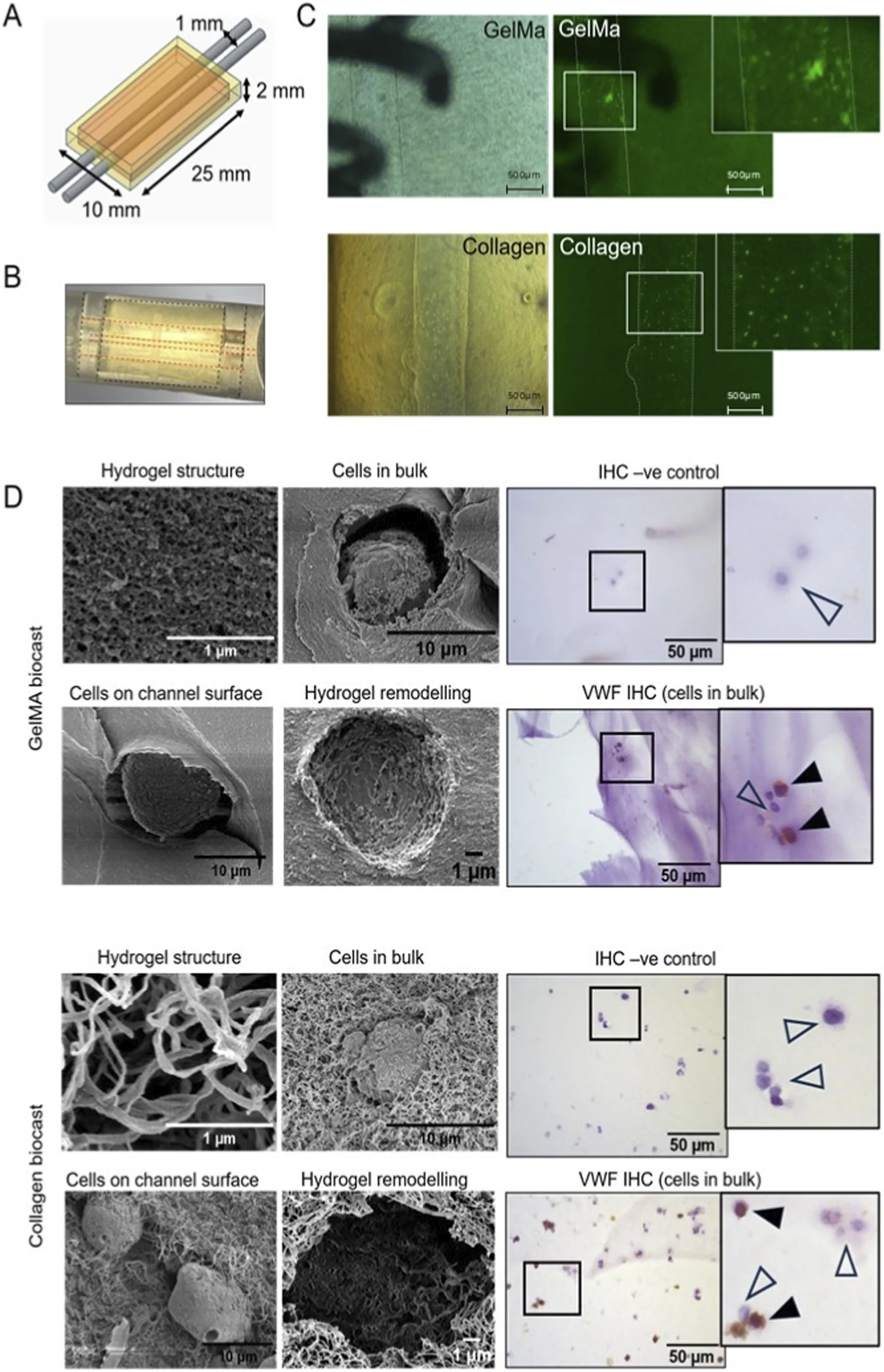
Biocasting approaches for validation and expansion of methodology. **(A)** Design for casting of GelMA and collagen cell-laden hydrogels with vascularisable channels. **(B)** A 3D printed mold used for casting and seeding of hydrogel chips, with a 2-channel chip inside, contained in a 2 mL microcentrifuge tube. **(C,D)** Brightfield (left) and epifluorescent images of casted HCT-116-laden GelMA (top) and collagen (bottom) 3D constructs with channels seeded with YFP-expressing HUVEC cells on day 1. **(D)** Structural characterisation and validation of cell-hydrogel contacts, hydrogel remodeling, and endothelial cell migration through the gel. Black arrowheads indicate positive IHC staining for the endothelial cell marker von Willebrand factor, while open arrowheads point to cells that are negative for VWF (i.e. HCT-116 cells).

The migration of HUVECs through three different materials (DLP printed GelMA, casted GelMA and casted collagen) was validated with an in-house transwell migration assay where the bottom of the transwell inserts was 3D biofabricated through the abovementioned methods and the media in the well contained VEGF to stimulate the transmigration through the gel (Supplementary Figure S1D).

With this set of experiments, we managed to reproduce and confirm the structure of the GelMA, the embedding of the cells in the channel lumen, the contacts with the substrate and the ability of the cells to remodel the hydrogel, as well as the migration of HUVECs within the bulk of the casted constructs (Figure 4D for casted constructs with channels; Supplementary Figure S1D for transwell migration assays with approximation of the migration distance). With regards to collagen biocasting, we observed similar cell growth and more facilitated migration, perhaps due to the fact that the hydrogel had higher porosity, which is consistent with lower protein (collagen) concentration and with the nature of the polymer that assembles into longer fibers relative to 10% GelMA and the nature of the polymer. Overall, these final experiments confirmed the feasibility and biocompatibility of the original designs for bioprinted (and biocasted) CRC models with channels that can be seeded with endothelial cells to mimic vascularisation.

## 4 Discussion

Angiogenesis together with invasion and metastasis are key hallmarks of cancer. Albeit very challenging, studying these processes *in vitro* is critical for our understanding of the mechanisms that underlie the interplay between tumor cells, endothelial cells and the ECM, and for the development of novel therapeutic approaches targeting blood vessel sprouting or intra- and extravasation of cancer cells.

Here, we test several approaches for biofabrication of vascularisable channels within cell-laden 3D constructs using DLP bioprinting. We focused on GelMA as it provides а suitable compromise between biocompatibility and mechanical properties and offers versatility in terms of biofabrication techniques (Mir et al., 2023). Importantly, we validated these designs and concepts by implementing (bio)casting using GelMA and type I collagen which also offers excellent biocompatibility together with sufficient mechanical and tensile strength (Mir et al., 2023). Consequently, this model and methodology also become accessible to research groups without specialised bioprinting equipment.

The blueprints for vascularised CRC models we present here bring an innovative approach expanding previously published work on endothelialised channels and bioprinting of cancer models (Supplementary Figure S4). There are only a handful of previous publications on GelMA bioprinted cancer models that include channels seeded with endothelial cells. Importantly, all of those reports employed extrusion-based (bio) printing. Nothdurfter et al. used a sacrificial hydrogel to create hollow channels seeded with endothelial cells and then micro-jet bioprinting with fibroblasts, MSCs or neuroblastoma spheroids for the bulk of their model (Nothdurfter et al., 2022). Shao et al. implemented coaxial printing with sacrificial hydrogel (gelatin) making up the lumen of the channels (with MDA-MB-231 breast cancer cells in the bulk of the model) (Shao et al., 2020). Ning et al. used GelMA for 3D printing (not 3D bioprinting with living cells) to create channels that were then seeded with HUVECs, while encapsulated neuroblastoma spheroids were added to the constructs after “endotheliasation”. Thus, it was demonstrated that culturing under dynamic conditions (with perfusion) would result in more metastatic behavior of cancer cells (Ning et al., 2022). Another example of an extrusion-based biofabrication method (rather than DLP bioprinting) to create channels is by sacrificial-free direct ink writing. Wu et al. also chose GelMA as the main component of their constructs, even though it was not the only polymer (like in our study), as it was combined with non-methacrylated gelatin, sodium alginate and collagen type I (respectively at 2,5%, 1,5%, 2% and 0,5% w/v) to create a vascularised perfusable hepatic metastasis model of neuroblastoma (Wu et al., 2024).

Besides the use of DLP-based bioprinting to create channels that can be subsequently vascularised, the other innovative aspect of this study is the actual composition of the hydrogel to support CRC cell growth (Wu et al., 2024). In terms of GelMA use for colorectal cancer modeling, a Pubmed search (“colorectal” AND “GelMA”) returned 20 results, of which only seven were loosely relevant to this work. These included GelMA-alginate microspheres with Caco-2 cells (Sanchez-Salazar et al., 2023), GelMA-dECM with embedded CRC cell on a microfluidic chip (Li et al., 2026), GelMA-alginate hollow constructs (using FRESH 3D printing) to support the seeding of CRC cells and fibroblasts (Tavares-Negrete et al., 2025), cell-laden GelMA directly casted into 96-well plates (Pamplona et al., 2024), GelMA-dECM scaffolds seeded with CRC cells (Liu et al., 2025), and 5%–7% GelMA dispensed into 96-well plates including HUVECs, fibroblasts and acute myeloid leukaemia cells to mimic vascular bed and macrophages (Adiguzel et al., 2026). However, none of these studies involved 3D bioprinting. The only example with 3D bioprinting we found was one using a GelMA-nanoclay hydrogel, which showed enhanced “stemness” properties of the CRC cells embedded in it (Zhang et al., 2022).

Several relevant papers have been published since the start of our experimental work. Moon et al. demonstrated the advantages of DLP bioprinting by combining GelMA (2%) with PEG (up to 5%) to create a perfusable liver model with HepG2 cells and channels seeded with HUVEC cells (Moon et al., 2025). The only other example of DLP-printing of GelMA channels that are seeded with endothelial cells in the context of cancer was again published in the end of 2025 by Siminska-Stanny et al. The authors focused on printability and different channel geometry comparing GelMA or GelMA-PEGDA DLP printed (not bioprinted) channels seeded with either HUVEC or osteosarcoma cells to demonstrate good adhesion along channels over 1 week (but without perfusion) (Siminska-Stanny et al., 2026). Kim et al. again from 2025 developed another DLP-printed GelMA based vascularised perfusable model. However, this work was with modified GelMA (with glycidyl methacrylate–GelMAGMA) and was not in the context of cancer, but rather the authors validated a functionalised endometrial (uterus) model (Kim et al., 2025).

In terms of DLP printing with GelMA specifically in the context of colorectal cancer, our literature search found only one publication. Even though Caco-2 cells were seeded on DLP printed GelMA constructs with different stiffness and geometry (Burmas et al., 2025), this work presents also the first DLP 3D bioprinted model of CRC using pure 10% GelMA.

It is important to note that this study has several limitations and technical challenges. Besides the actual handling of the constructs, loading the channels with a syringe and needle carries a risk of damaging the channel, or of contaminating the gel, both of which we encountered during this study. Nevertheless, we recognise that the main limitation in our experimental platforms is the lack of dynamic culturing/perfusion after bioprinting and endothelialisation. As shown in Figure 2 (top left), the design of our 3D bioprinted model allows for the connection of tubes through adapters and for perfusion through the channels. We would speculate, based on literature (Lovecchio et al., 2020; Elomaa et al., 2022), that this would support the growth of HUVECs over longer periods of time, which would provide the possibility for more long-term cancer modeling, but also for active drug delivery and measurement of response.

Another limitation is the lack of extensive experiments with 5% and 10% GelMA with different curing times, comparing bioprinted or biocasted data of gel stiffness that can affect cell proliferation and viability over time. Nevertheless, we have obtained rheology data. All formulations exhibited predominantly elastic, solid-like behavior, with loss-to-storage modulus ratios (G″/G′) of 8% for 5% GelMA and 3% for both 10% GelMA. These values are slightly below the ∼10% ratio often reported for native soft tissues. With regards to absolute stiffness, 5% GelMA (G′ ≈ 0.1 kPa) lies at the lower end of soft connective tissue ranges, 10% GelMA (G′ ≈ 1–2 kPa) overlaps with reported microvascular and perivascular environments (Humphrey et al., 2014), which makes this model physiologically relevant. Of note, due to the mechanical reinforcement of PEGDA, the GelMA-PEGDA hybrid showed a G’ of 7.23 kPa, which approaches values associated with small arterial walls requiring enhanced elastic stability (Humphrey et al., 2014). Nevertheless, the problem with biocompatibility and 3D bioprinting with 10% PEGDA in our hands could not be circumvented and this formulation was not exploited further in this study.

Compared to other studies (Nothdurfter et al., 2022; Wu et al., 2024), we used a relatively low concentration of cells (both HCT-116 and HUVECs) in our models. With regards to HCT-116 cells in the bulk of the gel, there are at least two important aspects that cell numbers may affect, printing accuracy being the first. The addition of cells in a resin could result in overcuring of unwanted regions due to light scattering or in underpolymerisation and reduced fidelity due to excessive light absorption (Neufeld et al., 2022). Both could lead to the narrower channels observed in our study (Supplementary Figure S1A). GelMA Swelling after several days of culturing is also possible (Zhu et al., 2019) despite the 10% GelMA high degree of substitution. Further curing time optimisation to exclude overpolymerisation and further material characterization studies on gelation kinetics, swelling ratio, and long-term degradation rates under culture conditions were, however, beyond the scope of this pilot study.

The second important aspect related to HCT-116 cells is their potential direct cell-cell interactions and indirect communication with HUVECs through secreted factors like VEGF (which HCT-116 have been shown to produce in high levels due to their *K-ras* mutation) (Okada et al., 1998; Ahluwalia et al., 2013). Therefore, it is plausible that the pro-agiogenic microenvironment that HCT-116 cells create can affect HUVECs survival and migration over time (without the addition of external VEGF). This, as well as more detailed investigation on whether HUVECs can form a functional endothelial barrier with tight junctions (that can be validated with immunostaining for CD31/VE-cadherin, Claudin and Occludin) and whether sprouting of endothelial cells can be observed over time, could not be carried out in the present study. Furthermore, GelMA remodeling by the cells suggested by SEM (Figures 2, 3D) could be explored in more detail by analyses of secreted matrix metalloproteinases (MMPs) that have readily available degradable sites within gelatin (and GelMA) (Kaemmerer et al., 2014). Thereby,we acknowledge that further optimisations of how different concentrations of cells would affect the structural properties of 10% GelMA, cell survival, growth and migration and more detailed characterisation of HUVECs and HCT-116 interactions with each other and with the matrix over time would be beneficial in the future, especially when this experimental system is tested under dynamic culturing conditions.

In summary, understanding of angiogenesis and metastasis, which are critical for cancer therapy outcome and patient overall survival, remains a challenge due to complex design of suitable experimental models. To address this need, we have designed a novel 3D bioprinted model of vascularised tumors that take advantage of two different biofabrication methods, biomaterials and cell types. The established methods and protocols here are widely accessible and reproducible and hold the potential to enable further studies on angiogenesis and metastasis. Enhancing the knowledge of these two processes through experimental models mimicking *in vitro* endothelial-cancer cell interactions represents an essential milestone towards development of future treatment strategies and survival rates.

## Data Availability

The original contributions presented in the study are included in the article/Supplementary Material, further inquiries can be directed to the corresponding authors.
