# Supplementary material for: Proof-of-concept validation and characterisation of a DLP 3D bioprinted vascularised colorectal cancer model
Source: Front Bioeng Biotechnol. 2026 Oct 1;14:1944341. doi: 10.3389/fbioe.2026.1944341 (PMC13669843; doi:10.3389/fbioe.2026.1944341)
Supplement: Supplementary file 1 [file Supplementaryfile1.docx]

Supplementary Material

Supplementary methods

Resin preparation and 3D bioprinting

For cell-free 3D printing for channel seeding optimisation, the resin consisting of 10% w/v GelMA supplemented with 10% v/v PEGDA (700 kDa), 10 mg/ml LAP and 1 mg/ml Tartrazine was used. PEGDA, while providing increased stiffness and better printability of more complex designs, was demonstrated to be highly cytotoxic in its non-crosslinked form (as confirmed by an MTT viability assay – data not shown) and was thus found unsuitable for bioprinting. Therefore, GelMA as the sole prepolymer in formulations was used for all further experiments. In GelMA-only formulations, a balance was struck between lowering the concentrations of LAP to decrease its cytotoxicity, lowering the concentration of Tartrazine to allow for sufficient polymerisation yielding strong enough gels, and finding the optimal crosslinking time to avoid excessive phototoxicity. The preparation methods of two batches of GelMA (proprietary protocols of MatriChem LTD) and the concentration of GelMA (5%, or 10%) were selected in a pilot bioprinting screening aiming to find the above-mentioned balance.

Printing was done on detachable silylated coverslips (developed and provided by MatriChem). In this approach, the surface of microscope glass coverslips is densely functionalised with methacrylic groups which enable the strong adhesion of the first 3D printed hydrogel layer to the glass surface by forming covalent bonds.

Confocal and electron microscopy

Hydrogel constructs with cells were fixed with PFA and both, cross-sections (~1 mm in thickness) and longitudinal slices of the constructs were prepared together with trans-well discs for detection and localization of YFP-expressing HUVEC cells either on the walls of the channels or in the bulk of the hydrogel. Labelling with phalloidin-TRITC was used to label all cells in the samples (HUVEC and HCT-116) (data not shown). Confocal microscopy was performed at IMCF-BIOCEV (Charles University, Prague) using Nikon CSU-W1. . For more thorough volumetric imaging, the remaining constructs were put on a Biotek Cytation C10 confocal microscope (MU Plovdiv) and Z-stacks (5 μm step) were obtained using a 10X objective. Due to light scattering, only about 500 μm of depth was clearly obtained (Supplementary Figure 1D).

The PFA fixed samples were first screened using 3D confocal microscopy to detect cells on the inner surface of the channels. Then longitudinal sections or cross sections through the channel were made by razor blade to expose the inner surface of the channels. Samples were then post-fixed with a 2.5% Glutaraldehyde and 3% Formaldehyde in 0.1 M PHEM followed by 1% osmium tetroxide in the same buffer. Samples were dehydrated in ethanol series and processed by critical point drying using Leica CPD300, mounted on pins, sputter-coated with a 5 nm platinum layer using a Leica EM ACE600 HV coater. Ultrastructural analysis was performed using a Helios NanoLab 660 G3 microscope (Thermo Fisher Scientific) at 2kV using secondary electrons detector.

Histological analysis and immunohistochemistry (IHC)

GelMA bioprinted and casted models, as well as collagen (bio)casted models were fixed in 10% neutral buffered formalin and embedded in paraffin following optimised in-house protocols. Briefly, the biofabricated models were stained in eosin for 30 seconds, dehydrated with 80%, 90%, 95% and absolute ethanol (total of 40 minutes), incubated in xylol for 5-15 minutes depending on the size (e.g. discs or channels), and were embedded in paraffin. Cross-sections (rather than longitudinal) of the vascularised models with channels were sliced at 6 µm thickness. Standard H&E staining was carried out to assess cell density and viability. IHC was carried out with a rabbit polyclonal anti-von Willebrand factor antibody (cat# A1335, AbClonal, USA) and a Leica universal kit based on peroxidase detection (cat # RE7110-CE, Leica, Germany).

Supplementary figures

**Supplementary Figure 1**

A) Bright field microscopy of 3D bioprinted channels (cross-section to the right showing the lumen of the channel) demonstrating the achievable printing resolution and approximate dimensions of the channels supported by the selected 10% GelMA resin containing live cells.
B) A representative picture of a 3D printed traswell insert with a 3D printed or casted insert at the bottom for transwell migration assays with HUVEC cells
C) A representative picture of a 3D DLP bioprinted 10% GelMA disc with HCT-116 cells
D) VEGF-induced migration in Transwell inserts. Left: Bioprinted GelMA. Middle: Cast GelMA. Right: Collagen. Volumetric reconstruction of a confocal microscopy Z stack (20x confocal objective, GFP fluorescence channel) from the seeding surface of the Transwells (cube edges highlighted in white, arrows show top-down direction when the discs were seeded). Scale bar (all images): 500 μm.
E) Confocal image of HUVEC cells seeded overnight into GelMA serpentine channels (10x magnification, 3D reconstruction of a Z-stack).

**Supplementary Figure 2 Rheology studies**

Rheological characterisation of the viscoelastic properties of three GelMA hydrogels – 5%, 10% and 20% w/v. A shear stress amplitude of 1 Pa was used to evaluate the storage modulus of the respective hydrogels (indicated in kPa). The error bars represent one SD from n=3.

**Supplementary Figure 3**

Histomorphological assessment of 3D biofabricated CRC models.

Comparison of cell morphology and viability between the three types of 3D biofabricated CRC models (DLP bioprinted or biocasted GelMA and biocasted collagen) via hematoxylin and eosin (H&E) staining demonstrating intact nuclei and cell density.

**Supplementary Figure 4**

PRISMA workflow for comparing our study to previously published work
